# Optimising Enzymatic Cross-Linking: Impact on Physicochemical and Functional Properties of Lupin Flour and Soy Protein Isolate

**DOI:** 10.3390/foods14111976

**Published:** 2025-06-03

**Authors:** Teguh Santoso, Yusur Al-Shaikhli, Thao M. Ho, Mishenki Rajapakse, Thao T. Le

**Affiliations:** 1 AUT Centre for Future Foods, Auckland University of Technology, Auckland 1010, New Zealand; 2 School of Science, Auckland University of Technology, Auckland 1010, New Zealand; 3 Department of Food and Nutrition, University of Helsinki, P.O. Box 66, 00014 Helsinki, Finland; 4 Helsinki Institute of Sustainability Science (HELSUS), University of Helsinki, P.O. Box 65, 00014 Helsinki, Finland; 5 HAMK Bio Research Unit, Häme University of Applied Sciences, P.O. Box 230, 13100 Hämeenlinna, Finland

**Keywords:** plant proteins, lupin flour, soy protein isolate, enzymatic cross-linking, laccase, transglutaminase, physicochemical properties, functional properties

## Abstract

The growing demand for plant-based protein alternatives has driven interest in protein modifications to enhance their functional properties in food applications. Enzymatic cross-linking using laccases derived from *Rhus vernicifera* (LR) and transglutaminase (TG) offers a promising strategy to enhance protein solubility, emulsifying properties, and foaming properties of food proteins. This study varied the enzymatic reaction conditions, including enzyme concentration, pH, temperature, incubation time, and ferulic acid addition, for the most effective cross-linking between proteins in lupin flour (LF) and soy protein isolate (SPI), resulting in changes in physicochemical and functional properties of the cross-linked proteins. LR-induced cross-linking in lupin and soy proteins was most favourable at 142.5 U/100 mg protein, pH 6, and 20 °C, where ferulic acid enhanced cross-linking efficiency with prolonged incubation (20 h). TG-induced cross-linking in lupin and soy proteins was most favourable at 1.25 U/100 mg protein, pH 6 and 30 °C, where high-molecular-weight aggregates were observed. Cross-linking modified protein surface characteristics, increasing ζ-potential and particle size due to protein aggregation, while ferulic acid further enhanced polymerisation. Morphological analysis revealed a porous powder structure across all samples with increased porosity in cross-linked samples as evidenced by the predominance of small fragments within the particles. Prolonged incubation led to partial disaggregation in LR-treated samples unless they were stabilised by ferulic acid. Under mild conditions (1 h, pH 6, 20 °C), LR and ferulic acid-added samples showed minor and significant improvements in protein solubility and foaming stability, respectively. Additionally, a significant increase in foaming ability was observed in ferulic acid-added LR samples after prolonged incubation (20 h), compared to the corresponding control. In contrast, prolonged incubation (20 h) or TG treatment had a lower foaming stability compared to the mild LR treatment. Emulsifying ability and emulsion stability showed limited variation across treatments. These findings suggest that cross-linking conditions influence specific functional properties, highlighting the need for further optimisation to achieve desired protein functionality in food applications.

## 1. Introduction

In recent years, plant proteins have gained increasing interest in the food industry, attributed to their diverse health benefits, sustainability, and abundance. However, their use in conventional human food remains limited due to the challenges in maximising their physicochemical and functional properties. Plant proteins tend to form large complexes or aggregates, which increases their molecular weight, making them less soluble and flexible than animal proteins [1]. Additionally, most plant proteins consist of several types of proteins with varying isoelectric points, making them more complex than single-protein systems. These variations in isoelectric points affect the functional properties of proteins, further limiting their effective use in food applications [2].

Therefore, it is important to investigate effective methods to improve the functional properties of plant proteins to ensure their successful use in food applications. Various strategies have been explored to enhance the functional properties of plant proteins, including physical methods, chemical modifications and biological methods [2]. These approaches operate through different mechanisms. Physical methods comprising both thermal and non-thermal treatments often induce protein unfolding, aggregation or even cross-linking [2,3,4]. Chemical modification methods, such as acylation or pH shifting, can result in cross-linking as well as structural changes. In addition, biological methods typically involve enzymatic processes, most notably enzymatic hydrolysis and enzymatic cross-linking [2].

Protein cross-linking is described as the process of joining protein molecules through intermolecular covalent bonds [5]. This process can create new macromolecular assemblies, which often lead to improved physicochemical and functional properties compared to the original proteins [5]. Cross-linking, particularly through enzymatic approaches, has become increasingly favoured as they are considered more natural than chemicals, require milder conditions, exhibit high specificity and are less likely to produce toxic by-products [6]. Moreover, enzymatic cross-linking enables targeted improvements in physicochemical properties such as ζ-potential, particle size distribution, and functional properties such as emulsifying, solubility, foaming properties, and gelling abilities, among others [7,8,9].

There are various commercially available enzymes capable of creating cross-linked proteins, yet they differ from each other in terms of cross-linking conditions and reaction mechanisms [10]. Laccase (EC 1.10.3.2) is a protein cross-linking enzyme that oxidises the phenolic moieties in proteins such as tyrosine residues, creating phenolic radicals. These phenolic radicals can further react with other phenolic radicals or free aromatic and amino groups present in proteins. As a result, different covalent linkages can be formed between protein side chains, as well as between proteins and small phenolic compounds [5,11]. Currently, only a few research studies have explored the efficacy of laccase-induced cross-linking on the functionality of plant-based proteins in food. Laccase-induced cross-linking reactions are reported to be enhanced by using ferulic acid. Ferulic acid is a phenolic compound widely used in various cosmetic and food applications, with the ability to increase the accessibility of reactive amino acids, thus improving the cross-linking process [12].

Transglutaminase (E.C. 2.3.2.13, TG) is one of the most common cross-linking enzymes and the only commercially available enzyme that is being used in the food industry [10]. TG catalyses an acyl-transfer reaction between the γ-carboxyamide group of glutamine residues and various primary amines, including lysine residues [10,13]. A recent study by Schlangen et al. [14] investigated the cross-linking effect of TG on pea protein and mung bean protein isolate, reporting that the use of TG can improve the mechanical and rheological properties of these protein isolate dispersions. Additionally, they noted that TG can form heteropolymer gels between different protein types, such as pea protein isolate and mung bean protein isolate, at high substrate concentrations. A study conducted by Herz et al. [15] demonstrated that the covalent cross-linking by TG enhances the functionality of heat-induced gels by acting as a functional binder, hence proving the ability of TG to significantly enhance the binding efficacy of soy protein isolate. Moreover, these findings suggest that TG could serve as a beneficial cross-linking agent for enhancing the functional properties of various plant proteins.

The effects of enzymes as cross-linking reagents on the physicochemical and functional properties of lupin and whey proteins were investigated in our previous research using two laccases, derived from *Rhus vernicifera* (LR) and *Trametes versicolor* (LT), and TG [7]. According to the results of the study, cross-linking with LR improved the overall functional properties, including protein solubility, emulsion stability, and foaming ability, while TG cross-linking slightly enhanced the emulsion stability and foaming stability of this plant-animal-based protein hybrid. These results indicate that the impact of cross-linking on physicochemical and functional properties varies depending on the type of enzyme and the proteins used.

Moreover, these enzymes’ activity largely depends on external conditions, such as pH and temperature. TG exhibits optimal activity between pH 5 and 8 but loses stability when exposed to temperatures above 70 °C. For fungal-derived laccases (e.g., LT), the optimal activity was best at a pH range of 4 to 5 on phenolic substrates and these enzymes exhibit stability within a temperature range of 30 to 60 °C [11,16]. Plant-derived laccase (e.g., LR) was reported to have higher optimum activity at pH 7 [17,18]. Laccases derived from fenugreek were reported to have an even lower optimum temperature at 20 °C [19]. Given these challenges, understanding the specific cross-linking conditions is crucial for optimising the use of these enzymes as cross-linking agents for plant proteins. While some studies have explored the use of TG and laccase on plant proteins [7,20,21], there is still limited understanding of the most effective cross-linking conditions for plant proteins such as lupin and soy. In particular, the effect of individual cross-linking conditions on the functional properties of these proteins has not been systematically studied.

Thus, the present study aimed to identify the most favourable or effective conditions for LR- and TG- induced cross-linking in a plant-based system (lupin flour and soy protein isolate) and to examine the effects of these conditions on the physicochemical (ζ-potential, particle size distribution, and morphology) and functional properties (protein solubility, emulsion properties, and foaming properties) of these protein mixtures. Factors examined include pH, incubation time, temperature, and enzyme concentrations. The impact of ferulic acid on LR cross-linking was also evaluated.

## 2. Materials and Methods

### 2.1. Materials

Lupin flour (LF, *Lupinus angustifolius*) and soy protein isolate (SPI) powder were obtained from Nothing Naughty (Tirau, New Zealand). The enzymes, ACTIVA^®^ Transglutaminase (TG) (100 U/g), were obtained from Ajinomoto Co. Inc. (Tokyo, Japan), while laccase (*Rhus vernicifera*, LR) enzyme (285 U/mg) was purchased from Sigma Aldrich (Darmstadt, Germany). Precision plus protein™ dual colour standards and Criterion™ TGX™ precast sodium dodecyl-sulphate polyacrylamide gel electrophoresis (SDS-PAGE) gels were purchased from Bio-Rad (Hercules, CA, USA). AccQ.Tag reagents were purchased from Apollo Scientific (Manchester, UK). Other chemicals and standards, including the amino acid standard (A9906, with the addition of asparagine and glutamine), acetic acid, aluminium sulphate, bromophenol blue, colloidal Coomassie blue G-250, citric acid, dithiothreitol (DTT), ethanol, ferulic acid, glycine, phosphoric acid, sodium dodecyl sulphate (SDS), tri-sodium citrate, tris hydrochloride (Tris-HCl), and urea, were also purchased from Sigma Aldrich (Darmstadt, Germany).

### 2.2. Proximate Analysis of Lupin Flour and Soy Protein Isolate

The proximate analysis of LF and SPI powder, including moisture, ash, protein, and fat contents, was determined by standardised methods as outlined in AOAC [22]. The moisture content was determined using AOAC method 934.01, ash content was measured following AOAC method 942.05, protein content was analysed by AOAC method 2001.11, and fat content was measured using AOAC method 2003.05. Carbohydrate content (%) was determined by subtracting the combined total content of moisture, ash, protein, and fat from 100%.

### 2.3. Enzyme Treatment of Lupin and Soy Protein

#### 2.3.1. Sample and Reagent Preparation

LF and SPI solutions were prepared by dispersing LF and SPI powder in Milli-Q water at 5% (*w*/*v*), with magnetic stirring at 400 rpm for 30 min at room temperature (25 °C). The pH of the solutions was adjusted to pH 4, 5, 6, 7, and 8 using 0.1 M NaOH or 0.1 M HCl. Finally, Milli-Q water was added to achieve a final concentration of 4% total powder weight (*w*/*v*) and mixed at a protein ratio of 1:1 before treatment.

LR solution was prepared by dissolving the LR powder in Milli-Q water to make up a concentration of 2.85 U/µL. TG solution was prepared by dissolving the TG powder in Milli-Q water to make up a concentration of 0.017 U/µL. The trans-ferulic acid powder was dissolved in Milli-Q water to make up a 25 mM ferulic acid solution. All solutions were vortexed for at least 1 min to ensure complete dispersion.

#### 2.3.2. Optimisation of Protein Cross-Linking Reactions Between LF and SPI

**Figure 1:**
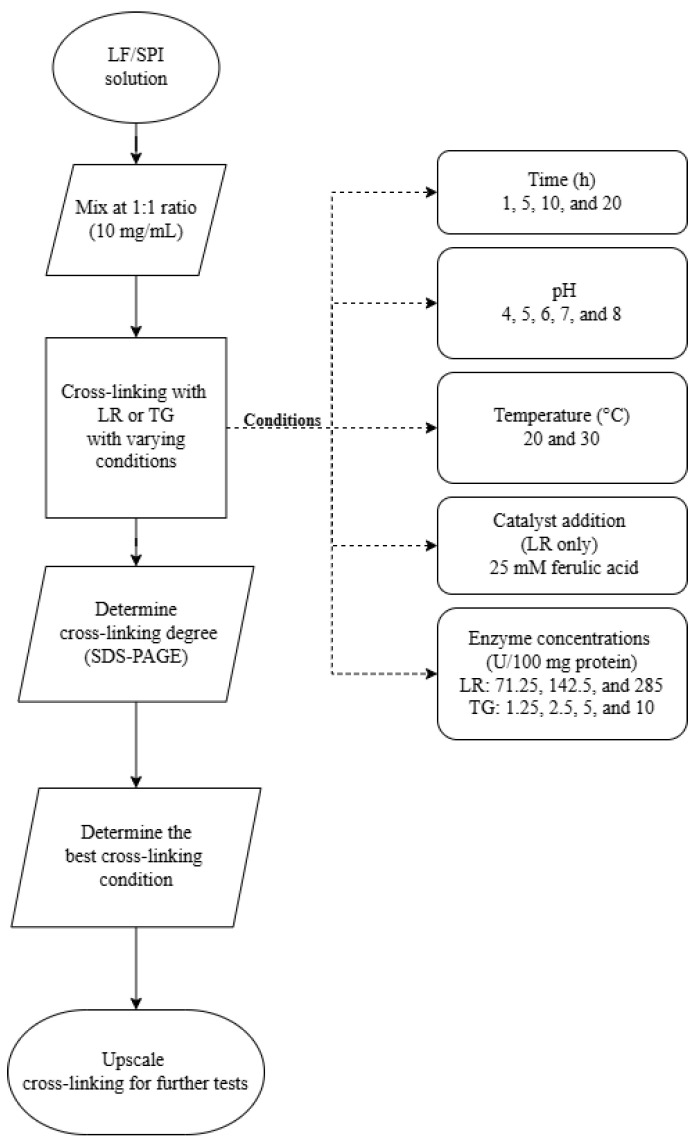
Flowchart of experimental design for optimising cross-linking conditions in lupin and soy (LS) mixtures. LF—lupin flour; SPI—soy protein isolate; LR—laccase; TG—transglutaminase.

Cross-linking reactions were conducted under varying conditions to investigate factors influencing enzymatic activity. These reactions were divided into three distinct experiments, each targeting a different variable. Figure 1 outlines the experiment design, and Table 1 details the conditions for the cross-linking:

- **Experiment 1 (incubation time):** Reaction time of 1, 5, 10, and 20 h at pH 7 and 30 °C.
- **Experiment 2 (pH variation):** Reactions at pH levels 4, 5, 6, 7, and 8 for 1 h at 30 °C.
- **Experiment 3 (ferulic acid addition, enzyme concentration, and incubation temperature):** Enzyme concentration ranged between 71.25, 142.5, and 285 U/100 mg protein for LR and 1.25, 2.5, 5, and 10 U/100 mg protein for TG. Samples were incubated for 1 h and 20 h at 20 °C and 30 °C at pH 6. Notably, ferulic acid (25 mM) was added as a catalyst in LR-treated samples.

The tested conditions were selected based on preliminary experiments and previously reported effective concentrations for similar protein systems [7,12,23]. After the optimisation experiments, the most effective cross-linking conditions were evaluated from each experiment. Then, an upscaled cross-linking experiment was performed.

#### 2.3.3. SDS-PAGE

The effects of different enzyme treatments were analysed using one-dimensional SDS-PAGE following a method adapted from Le et al. [24]. Protein samples were prepared by mixing with Laemmli sample buffer (62.5 mM Tris, 2% SDS, 25% glycerol, and 0.01% bromophenol blue) at a 1:4 (sample:buffer) ratio and reduced with 1 M DTT. The samples were then heated at 95 °C for 4 min. A 20 µL aliquot (containing 2 mg/mL of protein) was loaded on a precast SDS-PAGE gel (Criterion™ TGX™, Bio-Rad, Hercules, CA, USA). Electrophoresis was conducted at 200 V for 45 min using a running buffer (containing 25 mM Tris, 192 mM glycine, and 0.1% SDS). After electrophoresis, the gel was stained with colloidal Coomassie blue G-250 and destained in 1% acetic acid. The molecular weight (M_w_) of the protein bands was determined by comparing them to a molecular weight marker (Bio-Rad, Hercules, CA, USA), with a range of 10–250 kDa. Gel images were captured using the GelDoc Go imaging system (Bio-Rad, Hercules, CA, USA). The degree of cross-linking was assessed based on the distribution and intensity of the protein bands on the gel rather than by density measurements, as the cross-linked proteins were the primary focus.

### 2.4. Physicochemical Properties

Physicochemical properties, including ζ-potential, particle size, and morphology of cross-linked proteins, were determined following methods reported in our previous publication [7]. Here, a brief description of their principles was provided.

#### 2.4.1. ζ-Potential

The surface charge (ζ-potential) of the proteins was determined using a dynamic light scattering instrument (Zetasizer Nano ZS series, Malvern Panalytical Ltd., Malvern, UK). The protein dispersions (0.25%, *w*/*w*), after being homogenised under magnetic stirring (1000 rpm, 15 h), were diluted 1000-fold in Milli-Q water and then centrifuged (2000 rpm for 10 min) to remove undissolved particles. The supernatant of the centrifuged samples was taken for ζ-potential measurement (DTS1070 cell, Malvern Panalytical Ltd., Malvern, UK). Each sample was measured three times, with three ζ-potential readings recorded per measurement.

#### 2.4.2. Particle Size Distribution

The particle size of the proteins was determined using a Mastersizer Hydro 3000 SM integrated with a Hydro EV dispersion accessory (Malvern Instruments Ltd., Malvern, UK), with Milli-Q water as the dispersant. Before measurement, the proteins (1.0% *w*/*w*) were dispersed in Milli-Q water and stirred at 1000 rpm for 24 h. The refractive index of protein and the dispersant were set at 1.45 and 1.33, respectively. At least two measurements were conducted for each sample, with four readings per measurement.

#### 2.4.3. Morphology

The morphology of the powders was observed using a field emission scanning electron microscope (FESEM, S-4800, Hitachi, Tokyo, Japan) after they were coated with gold/palladium (208HR, Cressington Scientific Instruments, Watford, UK) to a thickness of 4 nm in two cycles. The coated samples were imaged under FESEM at 10 kV, 5 μA, with a 10 mm working distance. At least five different positions were imaged for each sample.

### 2.5. Functional Properties

#### 2.5.1. Protein Solubility

Protein solubility (%) was calculated as the ratio of soluble protein (from water extraction) to total protein (from urea and Tris-HCl “cocktail” extraction), expressed as a percentage, as outlined in our previous study [7]. The protein concentration in the water and “cocktail” extracts were determined using the Bradford assay [25].

For soluble protein extraction, samples were dispersed in Milli-Q water to make a 1% (*w*/*v*) concentration. The pH was then adjusted to pH 7 using 1 M NaOH or HCl. The mixture was stirred using a magnetic stirrer (200 rpm) for 30 min at 20 °C, followed by centrifugation (900× *g*, Gyrozen 1580R, Gyrozen, Gimpo, Republic of Korea) for 10 min at 20 °C, and the supernatant was used for soluble protein quantification.

For total protein extraction, the sample was dissolved in a cocktail (please refer to our previous publication [7] for cocktail composition) at the same concentration (1% *w*/*v*) and agitated at 600 rpm for 45 min at 20 °C using an orbital shaker (ISLD04HDG, Ohaus, Parsippany-Troy Hills, NJ, USA). The mixture was then centrifuged (1000× *g*, Gyrozen 1580R, Gyrozen, Gimpo, Republic of Korea) for 15 min at 10 °C, and the supernatant was used for total protein quantification.

#### 2.5.2. Emulsifying Properties

The emulsifying properties were analysed by following the method used by Ho et al. [26] with modifications. Powder samples were dissolved in Milli-Q water to make up a 1% concentration (*w*/*v*) and adjusted to pH 7 using 1 M NaOH or HCl. The solution was then stirred using a magnetic stirrer (200 rpm) for 30 min at room temperature (20 °C) and equilibrated overnight (20 h) at 4 °C. The following day, the samples were equilibrated to room temperature and a 5 mL sample solution was transferred to 15 mL centrifuge tubes and soybean oil was added at a 2:1 (sample:oil) ratio. An ultrasonic homogeniser equipped with a Φ10 probe (2BEM-150A, Bueno-Biotech, Nanjing, China) was used to create the emulsion. The instrument was set at 4 kHz with 80% amplitude for 45 s, with a pulsing sequence of 2.0 s pulse-on and 4.0 s pulse-off. The emulsions were then centrifuged (1100× *g*) for 5 min at 20 °C to separate excess oil. The emulsified layer was measured using a ruler to determine the emulsifying ability (EA). For emulsion stability (ES), the emulsion was heated for 30 min at 80 °C before centrifugation (1100× *g*) for 5 min at 20 °C. The EA and ES were calculated using Equations (1) and (2), respectively:

(1) $$\mathrm{EA}\left(\%\right)=\frac{\mathrm{Height}\;\mathrm{of}\;\mathrm{the}\;\mathrm{emulsified}\;\mathrm{layer}\;\mathrm{in}\;\mathrm{the}\;\mathrm{tube}\;\left(\mathrm{mm}\right)}{\mathrm{Height}\;\mathrm{of}\;\mathrm{the}\;\mathrm{total}\;\mathrm{content}\;\mathrm{in}\;\mathrm{the}\;\mathrm{tube}\;\left(\mathrm{mm}\right)}\times 100$$

(2) $$\mathrm{ES}\left(\%\right)=\frac{\mathrm{Height}\;\mathrm{of}\;\mathrm{the}\;\mathrm{emulsified}\;\mathrm{layer}\;\mathrm{after}\;\mathrm{heating}\;\mathrm{and}\;\mathrm{centrifugation}\;\left(\mathrm{mm}\right)}{\mathrm{Height}\;\mathrm{of}\;\mathrm{the}\;\mathrm{emulsified}\;\mathrm{layer}\;\mathrm{before}\;\mathrm{heating}\;\left(\mathrm{mm}\right)}\times 100$$

#### 2.5.3. Foaming Properties

The foaming properties were analysed by also following the method used by Ho et al. [26] and the same sample preparation as the emulsifying properties. To make the foams, sample solutions were mixed using a homogeniser (T-25 digital ULTRA-TURRAX, IKA, Selangor, Malaysia) at 25,000 rpm for 60 s at 20 °C. Foaming ability (FA) was determined by the percentage increase in the height of the foam layer (in mm) immediately after mixing, and foaming stability (FS) was determined by the percentage of the foam layer that remained after 30 min. The FA and FS were calculated using Equations (3) and (4), respectively:

(3) $$\mathrm{FA}\;\left(\%\right)=\frac{\mathrm{Height}\;\mathrm{of}\;\mathrm{the}\;\mathrm{foam}\;\mathrm{layer}\;\mathrm{in}\;\mathrm{the}\;\mathrm{tube}\;\left(\mathrm{mm}\right)}{\mathrm{Height}\;\mathrm{of}\;\mathrm{the}\;\mathrm{total}\;\mathrm{content}\;\mathrm{in}\;\mathrm{the}\;\mathrm{tube}\;\mathrm{before}\;\mathrm{foaming}\;\left(\mathrm{mm}\right)}\times 100$$

(4) $$\mathrm{FS}\;\left(\%\right)=\frac{\mathrm{Height}\;\mathrm{of}\;\mathrm{the}\;\mathrm{foam}\;\mathrm{layer}\;\mathrm{in}\;\mathrm{the}\;\mathrm{tube}\;\mathrm{after}\;30\;\min\;\left(\mathrm{mm}\right)}{\mathrm{Height}\;\mathrm{of}\;\mathrm{the}\;\mathrm{foam}\;\mathrm{layer}\;\mathrm{in}\;\mathrm{the}\;\mathrm{tube}\;\mathrm{at}\;0\;\min\;\left(\mathrm{mm}\right)}\times 100$$

### 2.6. Statistical Analysis

All statistical analyses were calculated using R (version 4.4.1). Differences between parameters were analysed using one-way analysis of variance (ANOVA). Tukey’s multiple comparison test was then applied (with a *p* < 0.05 threshold) to identify significant differences between the parameters. Results are reported as the mean values and standard deviation from at least triplicate measurements unless specified otherwise.

## 3. Results and Discussion

### 3.1. Proximate Analysis

The proximate composition of LF and SPI, including moisture, ash, fat, protein, and carbohydrate, are presented in Table 2. Comparing these two protein sources highlights the significant differences in their composition and potential impacts on their functional properties, especially for the protein, fat, and carbohydrate content. SPI contains significantly more protein compared to LF (87.6 and 43.70%, respectively), consistent with values reported in the literature [27,28,29,30]. This high protein content makes SPI an excellent ingredient for protein fortification in formulations. However, the removal of non-protein components during SPI processing results in considerably lower fat (0.09%) and carbohydrate (0.9%) content. In contrast, LF contains higher fat (6.00%) and carbohydrate (40.60%) content. These compositional differences influence the functional properties in various ways. SPI’s high protein content can enhance functional properties like foaming and gelling. However, the processing it undergoes can lead to protein aggregation, adversely affecting solubility [31]. On the other hand, LF’s higher fat and carbohydrate content can contribute to the emulsification properties and influence its protein solubility [30], making it more versatile for broader food applications. Beyond compositional differences, the pH also plays a crucial role in functional properties. SPI has a higher pH (7.72), whereas LF has a lower pH (6.19) (Table 2), comparable with the values found in literature [32,33]. pH influences solubility, surface charge, and structural conformation, which in turn affect functional properties such as emulsification, foaming, and gelation [34]. Therefore, understanding these intrinsic pH differences is essential for designing blended protein formulations, as pH adjustments could be necessary for better processing and functionality.

### 3.2. Effects of Reaction Conditions on Lupin and Soy Protein Cross-Linking

#### 3.2.1. Protein Profile of Lupin and Soy Proteins

**Figure 2:**
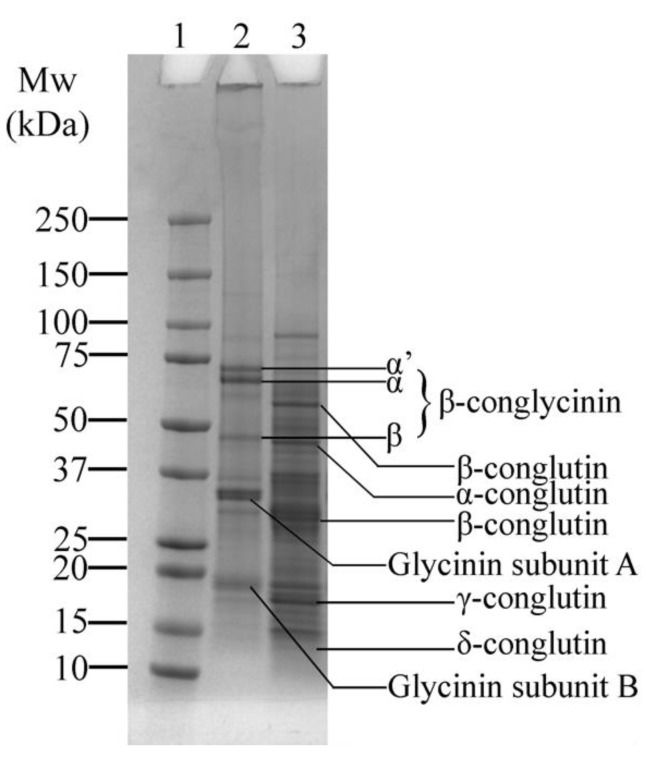
SDS-PAGE profile of soy protein isolate (SPI) and lupin flour (LF), showing the major protein bands: β-conglycinin and glycinin in SPI, and α, β, γ, and δ-conglutin in LF. Lane 1: Protein marker; Lane 2: SPI; Lane 3: LF.

The SDS-PAGE profile displays the protein composition of SPI and LF (Figure 2). Figure 2—Lane 2 shows a typical distribution of the protein bands of SPI corresponding to its major proteins, β-conglycinin and glycinin. β-conglycinin consists of three subunits, α’, α, and β, observed around ~73, ~69, and ~49 kDa, respectively. Glycinin, on the other hand, is composed of two subunits: the acidic A subunit (~35 kDa) and the basic B subunit (~19 kDa) [35]. Variations in the molecular weight were observed compared to previous studies [35,36,37,38], which could be attributed to differences in cultivars or extraction methods.

Lupin proteins also display the typical characteristic distribution, including globulins, which are comprised of α, β, γ, and δ-conglutin (Figure 2—Lane 3). The α-conglutin fraction was observed at around ~46 kDa, while β-conglutin appears as two distinct bands at ~65 kDa and ~32 kDa. Additionally, γ-conglutin was present at around ~18 kDa, consistent with previous findings [7,39,40]. Notably, smaller fragments of δ-conglutin, reported at ~12 kDa [40], are faintly visible, unlike in our previous study [7], where they were not present. This absence could be attributed to differences in cultivar or milder processing conditions of the LF used in this study.

#### 3.2.2. Effects of Varying Reaction Conditions on Lupin and Soy Protein Cross-Linking

##### Incubation Time

**Figure 3:**
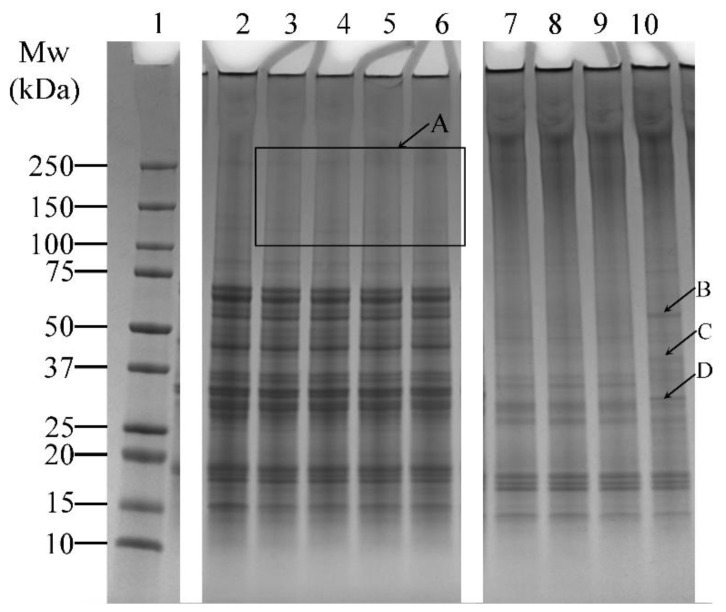
SDS-PAGE profile showing the cross-linking patterns of lupin and soy (LS) protein mixtures treated with laccase (LR) or transglutaminase (TG) at pH 7 and 30 °C under varying incubation times (1, 5, 10, and 20 h—H). Lane 1: Protein marker; Lane 2: LS-CT-1H Lanes 3–6: LS-LR-1H/5H/10H/20H; Lanes 7–10: LS-TG-1H/5H/10H/20H. CT—Control (non-enzyme-treated). A: Faint bands above 100 kDa in LR-treated samples, indicating limited cross-linking. B–D: New bands in TG-treated samples at 20 h, suggesting enhanced protein modifications. ***Summary:*** *TG treatment at 30 °C led to more visible cross-linking over time, with smearing and high molecular weight bands, unlike LR, which showed limited time-dependent enhancement.*

The effects of varying incubation times (1, 5, 10, and 20 h) on the cross-linking of lupin and soy proteins using LR and TG were examined (Table 1, Experiment 1). LR-treated samples (without ferulic acid) showed minimal visual differences across varying incubation periods (Figure 3, Lanes 3–6). Several faint bands above 100 kDa were observed (Figure 3, Box A), but no significant increase in band intensity was seen with extended incubation, suggesting limited enhancement of cross-linking under these conditions.

In contrast, TG-treated samples showed noticeable changes over time. Smearing between the 150–250 kDa region and additional bands above 250 kDa was prominent (Figure 3, Lanes 7–10), indicating the formation of high molecular weight protein aggregates. In addition, the intensity of lower molecular weight bands (10–75 kDa) decreased relative to the control sample (Figure 3, Lane 2), which also indicated the formation of protein aggregates. At 20 h of incubation, distinct bands appeared (Figure 3—Lane 10, arrows B, C, and D), suggesting that prolonged incubation may enhance modifications on the proteins, resulting in the formation of new bands.

##### pH

**Figure 4:**
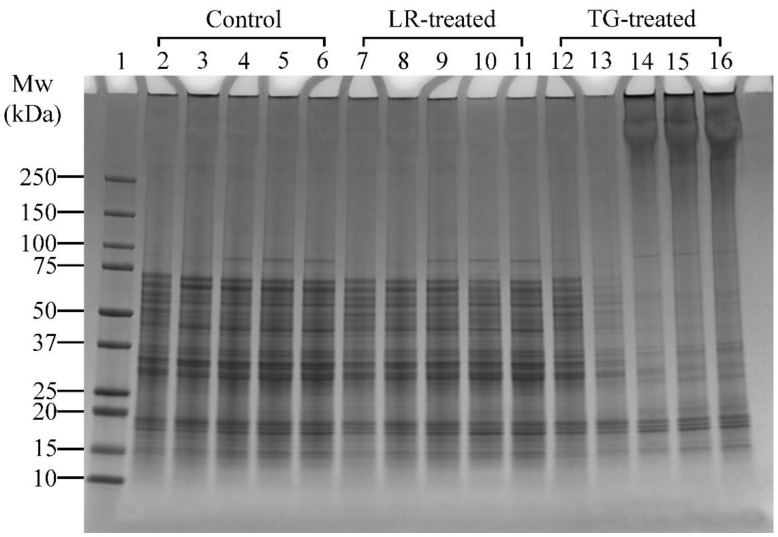
SDS-PAGE profile showing the cross-linking patterns of lupin and soy (LS) protein mixtures treated with laccase (LR) or transglutaminase (TG) for 1 h at 30 °C at varying pH (4, 5, 6, 7, and 8). Lane 1: Protein marker; Lanes 2–6: LS-CT pH 4–8 (controls); Lanes 7–11: LS-LR pH 4–8 (LR-treated); Lanes 12–16: LS-TG pH 4–8 (TG-treated). CT—Control (non-enzyme-treated). ***Summary:*** *TG-treated samples showed optimal cross-linking at pH 6 with dark smears and aggregates; LR treatments showed minimal changes across pH.*

A higher concentration of LR was applied in Experiment 2 (Table 1), which focused on pH effects, to enable a clearer visualisation of pH-dependent cross-linking compared to the time-based conditions in Experiment 1. Control samples treated at pH 6 and above showed a distinct band at ~85 kDa (Figure 4, Lanes 4–6). Similarly, LR- and TG-treated samples at pH 6 and above also showed distinct bands at ~85 kDa (Figure 4, Lanes 9–11 and Lanes 14–16, respectively). This observation indicated that the proteins at ~85 kDa were solubilised better at pH 6, 7, and 8. In LR-treated samples, no notable changes at high molecular weight regions (>250 kDa) were observed in control or LR-treated samples across all tested pH, suggesting limited protein cross-linking under these conditions. Therefore, pH 6 was chosen for further studies as it requires minimal pH modification.

Conversely, TG-treated samples clearly showed that cross-linking activity was better at pH 6, 7 and 8, as seen by the appearance of dark smears and protein aggregation around the wells (Figure 4, Lanes 14–16). At pH 4 and 5, minimal cross-linking was observed, with band disappearance at pH 5 (Figure 4, Lane 13), suggesting protein precipitation or reduced solubility at pH 5. At pH 6, monomer bands were lighter and high molecular weight (>250 kDa) aggregates were more prominent compared to pH 7 and 8 (Figure 4, Lane 14 vs. Lanes 15–16). This could indicate that most monomers were cross-linked at pH 6, suggesting that pH 6 is the most favourable condition for cross-linking with TG. Thus, among the tested conditions, pH 6 was selected for subsequent experiments for both LR and TG treatments, as it demonstrated the highest cross-linking efficiency, as shown by the presence of high-molecular-weight aggregates around the wells while requiring minimal pH adjustment.

##### Ferulic Acid Addition, Enzyme Concentration and Incubation Temperature

**Figure 5:**
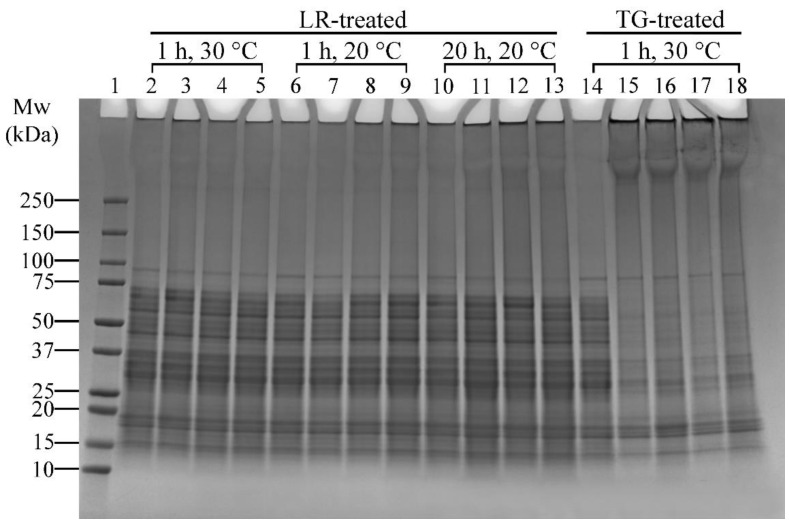
SDS-PAGE profile showing the effects of varying laccase (LR) (under the addition of ferulic acid—FeA), transglutaminase (TG) concentrations, and incubation temperatures on lupin and soy (LS) protein mixtures. Lane 1: Protein marker; Lane 2: LS-LR-285U-1H-30C; Lanes 3–5: LS-LR-71.25U/142.5U/285U-FeA-1H-30C; Lane 6: LS-LR-285U-1H-20C; Lanes 7–9: LS-LR-71.25U/142.5U/285U-FeA-1H-20C; Lane 10: LS-LR-285U-20H-20C; Lanes 11–13: LS-LR-71.25U/142.5U/285U-FeA-20H-20C; Lane 14: LS-CT-1H-30C; Lanes 15–18: LS-TG-1.25U/2.5U/5U/10U-1H-30C. CT—Control (non-enzyme-treated); U—Enzyme units (U/100 mg protein); H—Treatment time (h); C—Incubation temperature (°C). ***Summary:*** *LR cross-linking was most effective at 20 °C with 142.5 U/100 mg protein and ferulic acid; TG achieved extensive cross-linking even at low concentrations (1.25 U/100 mg protein) at 30 °C.*

The effects of ferulic acid on LR cross-linking were conducted in conjunction with enzyme concentration and incubation temperature effects (Table 1, Experiment 3). The SDS-PAGE results are presented in Figure 5.

##### Ferulic Acid and LR-Induced Cross-Linking

Ferulic acid has been shown to enhance LR-induced cross-linking [41]. In this study, ferulic acid was added to assess its effect under varying temperatures (20 °C and 30 °C) and incubation times (1 h and 20 h). There was minimal difference between samples without and with ferulic acid heated at 30 °C for 1 h, as evidenced by a slight increase in high molecular weight protein bands (>250 kDa) (Figure 5, Lanes 2 and 3–5, respectively). However, 20 °C and 1 h incubation conditions resulted in distinct protein aggregates around the well and slightly darker regions above 100 kDa or darker bands above 250 kDa (Figure 5, Lane 6 vs. Lanes 8–9), indicating enhanced cross-linking. The effect of ferulic acid was more pronounced in samples incubated at 20 °C for 20 h (Figure 5, Lane 10 vs. Lanes 11–13). These findings indicate that prolonged incubation amplifies ferulic acid’s effect on LR-induced cross-linking.

##### Enzyme Concentration and Incubation Temperature

The effects of LR concentrations (71.25, 142.5, and 285 U/100 mg protein) and temperatures (20 °C and 30 °C) on cross-linking reactions of lupin and soy proteins can be seen in Figure 5. Overall, there was less noticeable cross-linking in samples treated with 71.25 U of LR, and the reaction was enhanced when both 142.5 and 285 U of LR were used, particularly at 20 °C and 1 h treatment (Figure 5, Lane 7 vs. Lanes 8–9, respectively). The differences between these two concentrations were negligible, making 142.5 U/100 mg protein the most effective concentration for LR cross-linking under these conditions. Extending the incubation time to 20 h at 20 °C resulted in more pronounced protein aggregates around the well and darker regions above 100 kDa or darker bands above 250 kDa (Figure 5, Lanes 11–13), suggesting that longer incubation time enhanced LR-induced cross-linking efficiency. However, at 30 °C, increasing LR concentrations did not significantly enhance cross-linking, as indicated by the absence of distinct changes in band patterns (Figure 5, Lanes 3–5).

TG treatments were only tested at 30 °C based on preliminary tests and literature supporting its optimal activity at this temperature [23]. All TG-treated samples showed thick protein bands above 250 kDa and aggregations around the well (Figure 5, Lanes 15–18), suggesting effective cross-linking. The sample with the lowest enzyme concentration (1.25 U/100 mg protein, Lane 15) displayed cross-linking results comparable to those with higher concentrations (Lanes 16–18). This finding indicates that TG achieves extensive cross-linking even at lower enzyme concentrations, making 1.25 U/100 mg protein of TG the most effective concentration.

The most effective cross-linking conditions identified were then used in scaled-up reactions to generate sufficient material for further physicochemical and functional analysis (discussed in Section 3.3 and Section 3.4). The SDS-PAGE from the upscaled samples (Figure S1) showed that all treated samples have distinctly visible dark, thick bands at the top of the well, along with the formation of bands above 250 kDa, confirming that effective cross-linking occurred in the lupin and soy mixture in the upscaled study.

Overall, LR-induced cross-linking was most effective at 20 °C with 142.5 U/100 mg protein, particularly when ferulic acid was included and incubation time was extended to 20 h. In contrast, TG-induced cross-linking was optimised at 30 °C, with a lower concentration of 1.25 U/100 mg protein being sufficient for cross-linking. This observation can be attributed to the amino acid composition of the proteins and the specific mechanisms of action of each enzyme. Based on previous data, soy protein isolate contains relatively higher levels of lysine (3.4%) and glutamine (12.4%) compared to its phenolic amino acids, such as tyrosine (2.2%). Similarly, lupin flour has a similar composition of lysine (2.1%), glutamine (12.4%), and tyrosine (1.9%) [42]. These profiles suggest that there are more reactive sites available for TG-induced cross-linking (i.e., between glutamine and lysine residues) than for laccase-catalysed cross-linking (which targets phenolic residues like tyrosine). The relatively limited availability of phenolic amino acids may restrict the extent of cross-linking by laccase. Additionally, laccase has a broad substrate specificity as it can react with any phenolic compounds present in plant matrices [43], which may lead to non-specific reactions and reduce the efficiency of protein–protein cross-linking in plant-based systems. This could explain why TG treatment had more pronounced cross-linking effects in lupin and soy proteins in this study.

### 3.3. Physicochemical Properties

#### 3.3.1. ζ-Potential

**Figure 6:**
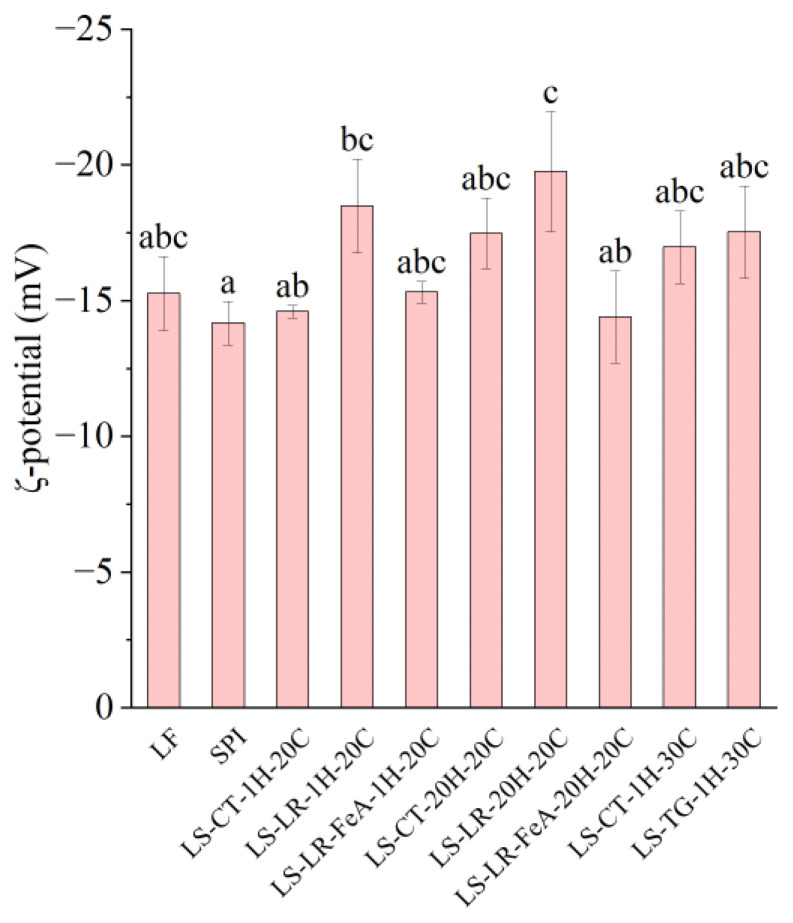
ζ-potential of lupin flour (LF), soy protein isolate (SPI), and mixtures of LF and SPI: control (LS-CT), laccase-treated (LS-LR), and transglutaminase-treated (LS-TG). FeA—Ferulic acid-added; H—Treatment time (h); C—Incubation temperature (°C). Different letters (a–c) indicate significant differences among samples (*p* < 0.05).

Both LR- (LS-LR-1H-20C and LS-LR-20H-20C) and TG (LS-TG-1H-30C)-treated samples increased the net negative charge (Figure 6), indicating protein cross-linking and/or modification between LF and SPI, despite their differing cross-linking mechanisms. LR catalyses oxidative cross-linking, while TG forms covalent bonds between glutamine and lysine residues [5,10]. Specifically, compared to untreated samples (LS-CT-1H-20C), laccase-treated LS-LR-1H-20C exhibited a higher absolute ζ-potential (−14.61 vs. −18.50 mV, respectively), similar to that of TG-treated LS-TG-1H-30C (−17.54 mV). A higher negative charge can enhance emulsifying stability through increased electrostatic repulsion, but protein solubility remains a key factor in emulsification [44]. The impacts of enzymatic cross-linking on the surface charge depended on the cross-linking conditions. The addition of ferulic acid influences the ζ-potential by reducing its negativity (LS-LR-FeA-1H-20C: −15.32 mV vs. LS-LR-1H-20C: −18.50 mV, and LS-LR-FeA-20H-20C: −14.40 mV vs. LS-LR-20H-20C: −19.77 mV), indicating that ferulic acid may mask some negative charges through covalent or non-covalent interactions with proteins [21]. Regardless of the presence of LR and ferulic acid, increasing the treatment time did not significantly affect the surface charge (LS-LR-1H-20C: −18.50 mV vs. LS-LR-20H-20C: −19.77 mV, and LS-LR-FeA-1H-20C: −15.32 mV vs. LS-LR-FeA-20H-20C: −14.40 mV).

#### 3.3.2. Particle Size Distribution

**Figure 7:**
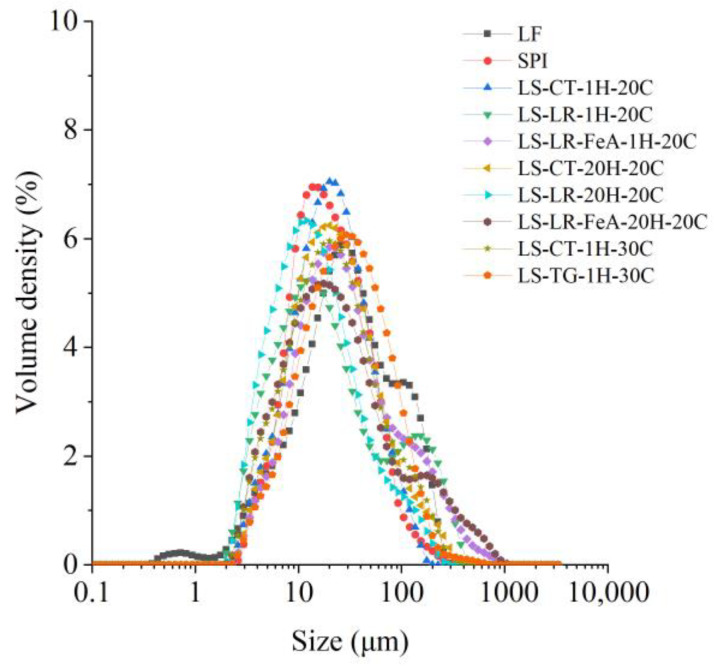
Particle size distribution of lupin flour (LF), soy protein isolate (SPI), and mixtures of LF and SPI: control (LS-CT), laccase-treated (LS-LR), and transglutaminase-treated (LS-TG). FeA—Ferulic acid-added; H—Treatment time (h); C—Incubation temperature (°C).

LR-treated samples exhibited larger D[4,3] values than non-enzyme-treated ones, with LS-LR-1H-20C (47.8 µm) showing a significantly larger particle size than LS-CT-1H-20C (28.2 µm) (Figure 7 and Table 3), suggesting that LR-induced oxidative cross-linking promoted aggregation. The addition of ferulic acid (LS-LR-FeA-1H-20C) further increased D[4,3] to 58.3 µm, indicating enhanced polymerisation through radical-mediated reactions. Similarly, TG treatment also increased particle size, as LS-TG-1H-30C exhibited a D[4,3] of 42.4 µm, larger than LS-CT-1H-30C (35.9 µm), suggesting that TG-mediated covalent cross-linking promoted protein aggregation. Additionally, LS-TG-1H-30C had the highest D[3,2] (17.9 µm), indicating the formation of a more compact network compared to LR-treated and non-enzyme-treated samples.

Increasing incubation time from 1 h to 20 h reduced D[4,3] in LR-treated samples (LS-LR-1H-20C: 47.8 µm → LS-LR-20H-20C: 24.7 µm). This suggests possible network rearrangement or disaggregation over time. However, when ferulic acid was present (LS-LR-FeA-20H-20C), D[4,3] remained high (61.2 µm), implying that ferulic acid-supported cross-links were more stable against breakdown. The supporting role of ferulic acid in cross-linking, seen on the SDS-PAGE gel (Figure 5, Lanes 6–13), was discussed in Section 3.2.2.

#### 3.3.3. Morphology

**Figure 8:**
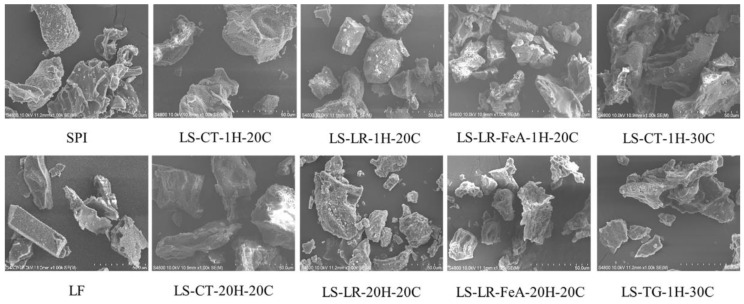
Morphology of lupin flour (LF), soy protein isolate (SPI), non-enzyme-treated LF and SPI mixture (LS-CT), and enzyme-treated mixtures with laccase (LS-LR) and transglutaminase (LS-TG) at 20 °C and 30 °C, respectively. FeA—Ferulic acid-added; H—Treatment time (h); C—Incubation temperature (°C).

The morphology of all samples was similar, characterised by irregularly shaped particles with rough edges and non-uniform forms (Figure 8). There was a significant variation in particle size, with some appearing larger and more intact, while others were smaller and more fragmented. Some particles displayed a plate-like, layered structure, while others appeared aggregated or clustered together, further highlighting the diversity in morphology. These are common characteristics of freeze-dried powders [45]. Additionally, the results demonstrated that enzyme cross-linking, irrespective of the treatment duration and the presence of ferulic acid (i.e., LS-LR-FeA-1H-20C, LS-LR-20H-20C, LS-LR-FeA-20H-20C, and LS-TG-1H-30C) led to a more porous powder structure. This increased porosity was evidenced by the predominance of small fragments within the particles, suggesting a disruption or modification in their microstructure during freeze-drying. Similar findings have been reported for chickpea protein cross-linked by TG, where enzyme cross-linking enhanced the porous structure [23].

### 3.4. Functional Properties

#### 3.4.1. Protein Solubility

**Figure 9:**
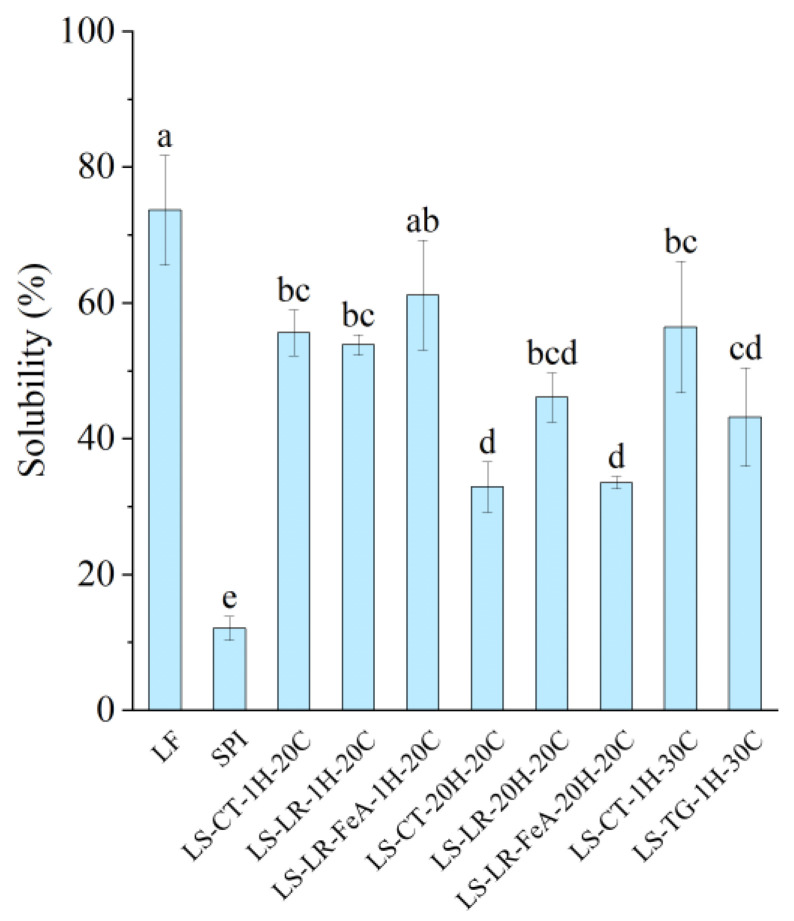
Protein solubility of lupin flour (LF), soy protein isolate (SPI), non-enzyme-treated LF and SPI mixture (LS-CT), laccase-treated mixture (LS-LR), and transglutaminase-treated mixture (LS-TG). FeA—Ferulic acid-added; H—Treatment time (h); C—Treatment temperature (°C). Different letters (a–e) indicate significant differences among samples (*p* < 0.05).

The control sample, LF, exhibited the highest solubility at 73.47%, while SPI had the lowest solubility at 12.14% (Figure 9), which could be attributed to the higher degree of protein aggregation associated with SPI’s production [31]. In a mixed system, the protein solubility of the LS mixture varied depending on incubation time. Samples treated for 1 h had a higher solubility (53–61%), while samples treated for 20 h had a lower solubility (32–46%). Based on our previous study, the solubility of the control mixture was also relative to the difference between the two proteins [7]. Therefore, as the mixture is at a 1:1 ratio, the solubility was predicted to be approximately 43%. However, the recorded solubility was both higher and lower than predicted; thus, it can be stipulated that protein interaction and time factors may influence protein solubility.

Comparing the enzyme-treated samples, those treated with LR and TG displayed varying solubility levels. Notably, LS-LR-1H-20C (53.70%) showed similar solubility to its control (LS-CT-1H-20C: 55.48%). The sample treated with ferulic acid and incubated for 1h (LS-LR-FeA-1H-20C) demonstrated slightly improved solubility compared to the non-ferulic acid-added sample (LS-LR-1H-20C) at 61.04% and 55.48%, respectively, possibly due to ferulic acid’s ability to improve cross-linking [12]. However, these differences were not statistically significant, indicating that short-term LR treatment had a limited impact on solubility. Similarly, TG-treated samples showed reduced solubility compared to their controls (LS-TG-1H-30C: 43.73% vs. LS-CT-1H-30C: 56.27%). A study conducted by Nivala et al. [45] reported a similar observation where TG treatment led to decreased solubility of fava bean protein isolate. However, the authors also reported improved TG-treated oat protein isolate at low enzyme concentrations.

Extending the incubation to 20 h decreased solubility, reflecting the negative influence of prolonged heating on the protein system. The solubility of LS-CT-1H-20C was significantly higher than that of LS-CT-20H-20C (55.48% vs. 32.96%). Similarly, LS-LR-1H-20C showed higher solubility compared to LS-LR-20H-20C (53.70% vs. 46.10%), although the difference was not statistically significant. The ferulic acid-added sample incubated for 20 h (LS-LR-FeA-20H-20C) also had significantly lower solubility (33.62%) compared to its 1 h counterpart (LS-LR-FeA-1H-20C: 61.04%).

As discussed in our previous study [7], proteins with higher absolute ζ-potential tend to exhibit improved solubility due to stronger interactions with dipolar water molecules. For instance, LS-LR-20H-20C had a higher absolute ζ-potential than LS-LR-FeA-20H-20C (−19.77 mV vs. −14.40 mV) and showed a corresponding trend in protein solubility (46.10% vs. 33.62%, respectively). However, this relationship was not consistently observed across all samples. Specifically, SPI and LS-LR-FeA-20H-20C had similar ζ-potential (−14.18 mV vs. −14.40 mV) but significantly different protein solubility (12.14% vs. 33.62%, respectively). This discrepancy may be attributed to SPI’s extensive thermal processing, which likely induced protein aggregation, a known factor to reduce solubility despite high surface charge [40]. These observations suggest that while ζ-potential can influence solubility, other factors such as protein aggregation and structural rigidity induced by processing effects also play a critical role.

#### 3.4.2. Emulsifying Properties

##### Emulsifying Ability

**Figure 10:**
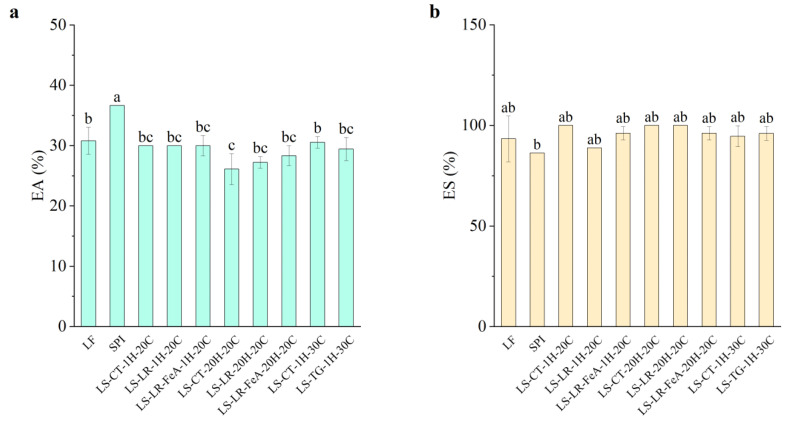
Emulsifying ability (**a**) and emulsion stability (**b**) of lupin flour (LF), soy protein isolate (SPI), non-enzyme-treated LF and SPI mixture (LS-CT), laccase-treated mixture (LS-LR), and transglutaminase-treated mixture (LS-TG). FeA—Ferulic acid-added; H—Treatment time (h); C—Treatment temperature (°C). Different letters (a–c) indicate significant differences among samples (*p* < 0.05).

The emulsifying ability of the samples showed small variation across treatments (Figure 10a). LF exhibited an emulsifying ability of 30.81%, while SPI had a significantly higher emulsifying ability of 36.67%. This result aligns with expectations, as SPI’s higher protein content likely provides more surface-active molecules, enhancing emulsifying ability. Enzyme treatments, including LR and TG, showed no significant change in emulsifying ability compared to the control samples. LR-treated samples at 1 h (LS-LR-1H-20C) and the control samples (LS-CT-1H-20C) exhibited emulsifying ability values of 30%, while TG-treated samples at 1 h (LS-TG-1H-30C) and the control samples (LS-CT-1H-30C) exhibited emulsifying ability values of 29.44% and 30.56%, respectively.

Additionally, the addition of ferulic acid did not significantly alter emulsifying ability. Samples treated with ferulic acid for 1 h showed consistent emulsifying ability values (30.00%) compared to those without ferulic acid. However, a slight improvement was observed in samples treated for 20 h, with the emulsifying ability of the control being 26.11% (LS-CT-20H-20C), LR-treated sample without ferulic acid (LS-LR-20H-20C), 27.22%, and LR-treated sample with ferulic acid (LS-LR-FeA-20H-20C), 28.33%. The result suggests that the addition of ferulic acid to the treatment may enhance emulsifying ability. This result differs from our previous study [7], in which treated samples showed reduced emulsifying ability under similar conditions. This difference is likely due to the different substrates used, suggesting that optimisation may be required for each substrate. TG-treated samples (LS-TG-1H-30C) exhibited similar results to the previous study, where a small decrease in emulsifying ability was observed compared to the control (LS-CT-1H-30C) at 29.44% and 30.56%, respectively. The difference was not statistically significant, indicating that these treatments do not severely compromise emulsifying ability. While the conditions in this study were insufficient for definitive conclusions about enzyme treatments, the findings provide valuable baseline data for future studies.

##### Emulsion Stability

Emulsion stability across all samples was consistently high, with values close to or exceeding 100% and no significant differences were observed between enzyme-treated and control samples (Figure 10b). This observation is likely due to the complexity of plant materials and processing effects, which may naturally stabilise emulsions. As such, SPI’s lower emulsion stability (86.36%) compared to the other samples may be attributed to its minimal carbohydrate content (0.90%), which could negatively impact SPI’s emulsion stability. In contrast, control and enzyme-treated samples, e.g., LS-CT-1H-20C, LS-CT-20H-20C, and LS-LR-20H-20C, showed higher emulsion stability, with some reaching 100% emulsion stability.

Given the lack of statistically significant differences, it is difficult to directly link changes in emulsion stability to enzymatic treatment. Although enzymatic cross-linking is known to potentially improve stability by limiting coalescence and phase separation [12,41,46], these effects could not be confirmed in the present study. Further investigation is required to better understand the mechanisms influencing emulsion stability in this plant-based protein system.

#### 3.4.3. Foaming Properties

##### Foaming Ability

**Figure 11:**
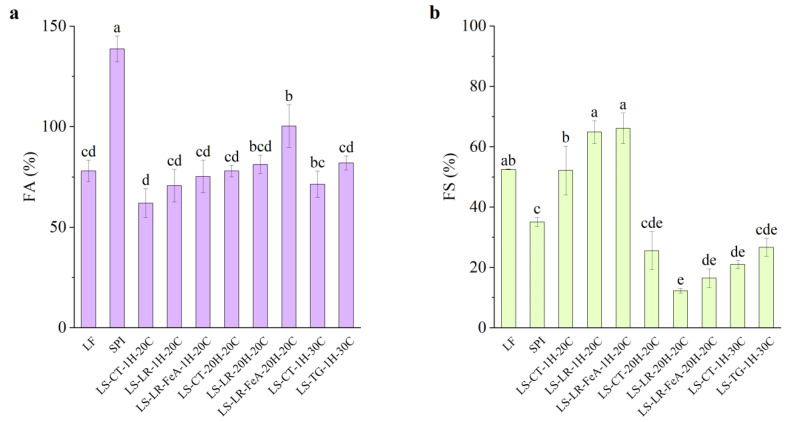
Foaming ability (**a**) and foaming stability (**b**) of lupin flour (LF), soy protein isolate (SPI), and mixtures of LF and SPI: control (LS-CT), laccase-treated (LS-LR), and transglutaminase-treated (LS-TG). FeA—Ferulic acid-added; H—Treatment time (h); C—Treatment temperature (°C). Different letters (a–e) indicate significant differences among samples (*p* < 0.05).

The foaming ability of SPI was significantly higher compared to all other samples (138.67%), attributed to its high protein content (Figure 11a). In contrast, LF exhibited a lower foaming ability at 78.00%. Furthermore, LS mixture samples treated at 20 °C for 1 h (LS-CT-1H-20C) showed a further decrease in foaming ability to 62.00%. This reduction compared to LF (78.00%) and SPI (138.67%) may be attributed to protein–protein interactions between LF and SPI upon mixing. These interactions could hinder interfacial adsorption, resulting in reduced foam formation. Additionally, the higher carbohydrate and fibre content in LF flour may have contributed to the reduced interfacial activity and foam formation [47]. Interestingly, the foaming ability of the control samples varied depending on the treatment conditions. Compared to the 20 °C 1 h control (62.00%), samples treated at 30 °C for 1 h (LS-CT-1H-30C) exhibited an increased foaming ability of 71.36%, while samples treated at 20 °C for 20 h (LS-CT-20H-20C) had an even higher foaming ability of 78.00%.

Regarding the enzyme-treated samples, both LR and TG treatments resulted in an improvement in foaming ability. LR-treated samples for 1 h showed slight but statistically insignificant improvements, with foaming ability increasing from 62.00% (LS-CT-1H-20C) to 70.67% (LS-LR-1H-20C) and 75.33% (LS-LR-FeA-1H-20C). Similarly, TG-treated samples improved from 71.36% to 82.00% (LS-CT-1H-30C to LS-TG-1H-30C, respectively). In addition, ferulic acid-added LR-treated samples for 20 h exhibited a significant increase in foaming ability, rising from 78.00% (LS-CT-20H-20C) to 81.33% (LS-LR-20H-20C) and 100.37% (LS-LR-FeA-20H-20C). These observations align with our previous study [7] and a study by Liu et al. [48], who demonstrated improved foaming ability in SPI due to cross-linking.

More importantly, the positive trend observed with the addition of ferulic acid suggests that it enhances LR treatment. This finding indicates that the treatment conditions used in this study are promising for the current application but require further optimisation. These findings highlight the importance of substrate and enzyme synergies.

##### Foaming Stability

Foaming stability varied depending on the protein source. LF, with its complex substrate, exhibited higher foaming stability (52.50%) compared to SPI (35.10%). The relatively high foaming stability in LF may be partially attributed to the presence of carbohydrates, which can stabilise air–water interfaces and improve foam retention [47]. However, the carbohydrate and fibre contents were not directly quantified in this study. Future work should include their measurement to better understand their role in modulating functional properties.

Enzyme-treated samples showed distinct trends in foaming stability. For LR-treated samples at 20 °C for 1 h, foaming stability increased from the control 52.23% (LS-CT-1H-20C) to 64.93% (LS-LR-1H-20C) and 66.10% (LS-LR-FeA-1H-20C) for LR without and with the addition of ferulic acid, respectively. Although a significant difference was observed between the control and LR-treated samples, the difference between samples with and without ferulic acid was not statistically significant. Conversely, LR-treated samples at 20 °C for 20 h exhibited a decreasing trend, with foaming stability dropping from 25.55% (LS-CT-20H-20C) to 12.33% (LS-LR-20H-20C) and 16.53% (LS-LR-FeA-20H-20C). However, the addition of ferulic acid has a higher foaming stability than non-ferulic acid added samples, suggesting that ferulic acid may offer some protective effect against structural breakdown during extended treatment.

TG-treated samples exhibited a slight increase in foaming stability from 20.95% to 26.73% (LS-CT-1H-30C and LS-TG-1H-30C, respectively), but the improvement was not statistically significant, suggesting that the treatment condition may not favour foaming stability. However, the upward trend is consistent with the findings of Xiang et al. [49], who reported significant improvements in both foaming ability and stability of SPI treated with TG at 4 U/g for 45 min. The difference in enzyme concentration and processing conditions used in the present study may explain the comparatively limited effects observed.

Overall, foaming stability decreased with prolonged incubation (12.33% at 20 °C for 20 h, LS-LR-20H-20C) and with TG treatment (26.73% at 30 °C for 1 h, LS-TG-1H-30C), compared to the higher stability observed in mildly LR-treated samples (64.93% at 20 °C for 1 h, LS-LR-1H-20C). This suggests that extensive processing and cross-linking may reduce protein flexibility or interfacial activity in the foam system. This trend is partially reflected in the larger particle size observed in prolonged ferulic acid-added LR- and TG-treated samples, which corresponded with lower foaming stability (LS-LR-FeA-20H-20C: 61.2 µm, 16.53%; LS-TG-1H-30C: 42.4 µm, 26.73%). However, this relationship was not consistent across all samples. For example, the mildly treated LR sample (LS-LR-1H-20C), despite having a similarly large particle size (47.8 µm), exhibited significantly higher foaming stability (64.93%). Conversely, prolonged LR-treated (LS-LR-20H-20C) resulted in the smallest particle size (24.7 µm) but had the lowest foaming stability (12.33%). This observation suggests that the extent and nature of cross-linking, rather than particle size alone, may be the dominant factor. This pattern aligns with our previous study [7], where extensive cross-linking was associated with a decrease in foaming properties. Additionally, the complex nature of plant protein matrices may contribute to this behaviour. Commercial SPI, for example, also showed relatively low foaming stability compared to LF and mildly LR-treated LS samples. Further investigation is needed to clarify these mechanisms, particularly the interactions between matrix complexity, cross-linking degree, and interfacial behaviour.

## 4. Conclusions

The study presents the first exploration of LR and TG cross-linking conditions such as incubation time, pH, temperature, enzyme concentration, and the presence of ferulic acid, on the degree of lupin and soy protein cross-linking, as well as the impact of certain conditions on the physicochemical and functional properties of these plant-based proteins.

For LR, the most effective cross-linking was achieved at 142.5 U/100 mg protein, pH 6, and 20 °C, where ferulic acid significantly enhanced the extent of cross-linking, particularly after 20 h of incubation. TG cross-linking was most effective even under the mildest conditions tested (1.25 U/100 mg protein, pH 6, 30 °C for 1 h). Both LR and TG treatments modified protein surface characteristics, as shown by increased absolute ζ-potential and particle size due to aggregation. Ferulic acid further enhanced polymerisation in LR-treated samples. Morphological analysis revealed increased porosity and fragmentation in all cross-linked powders, suggesting structural disruption during freeze-drying.

Functionally, short-term LR cross-linking with ferulic acid modestly improved solubility and foaming stability, though the improvements were not always statistically significant. A significant increase in foaming ability was observed in ferulic acid-added LR samples after prolonged incubation (20 h), compared to the corresponding control. In contrast, prolonged LR incubation or TG treatment had a lower foaming stability compared to the mild LR treatment. Meanwhile, emulsifying ability and emulsion stability showed limited changes across treatments.

Overall, these findings indicate that cross-linking conditions influence protein functionality. Given the varying results and the complexity of plant-based protein systems, future work should investigate molecular-level mechanisms using advanced techniques such as circular dichroism, FTIR, and mass spectrometry. In addition, statistical optimisation tools such as response surface methodology (RSM) should be applied to fine-tune processing conditions for targeted food applications.

## Figures and Tables

**Table 1:** Experimental conditions used to optimise cross-linking reactions of lupin and soy proteins.

Experiments	Variation	Time (h)	pH	Temperature (°C)	Catalyst	Enzyme Concentration (U/100 mg Protein)
Experiment 1	Incubation time	1, 5, 10, and 20	7	30	-	LR: 142.5 TG: 10
Experiment 2	pH	1	4–8	30	-	LR: 285 TG: 10
Experiment 3	Ferulic acid addition, enzyme concentration, and incubation temperature	1 and 20	6	20 and 30	25 mM ferulic acid (LR only)	LR: 71.25, 142.5, and 285 TG 1.25, 2.5, 5, and 10

**Table 2:** Proximate analysis of lupin flour and soy protein isolate powder.

Attributes	LF	SPI
Moisture (%)	7.10 ± 0.30	6.50 ± 0.10
Ash (%)	2.60 ± 0.04	4.90 ± 0.02
Fat (%)	6.00 ± 0.02	0.09 ± 0.03
Protein (%)	43.70 ± 0.90	87.60 ± 0.30
Carbohydrate (%)	40.60 ± 0.80	0.90 ± 0.20
pH	6.19 ± 0.02	7.72 ± 0.02

**Table 3:** Particle size of lupin flour (LF), soy protein isolate (SPI), and mixtures of LF and SPI: control (LS-CT), laccase-treated (LS-LR), and transglutaminase-treated (LS-TG), including volume-based diameter (D[4,3]) and surface area-based diameter (D[3,2]). FeA—Ferulic acid-added; H—Treatment time (h); C—Incubation temperature (°C).

Sample Name	D[4,3], µm	D[3,2], µm
LF	46.2 ± 6.2 ^d^	12.4 ± 0.5 ^f^
SPI	27.9 ± 4.4 ^d^	14.3 ± 0.6 ^d^
LS-CT-1H-20C	28.2 ± 0.3 ^d^	14.1 ± 0.1 ^de^
LS-LR-1H-20C	47.8 ± 1.9 ^b^	11.4 ± 0.1 ^g^
LS-LR-FeA-1H-20C	58.3 ± 6.4 ^a^	17 ± 0.1 ^b^
LS-CT-20H-20C	36.4 ± 5.1 ^c^	15 ± 0.7 ^c^
LS-LR-20H-20C	24.7 ± 0.5 ^d^	9.9 ± 0 ^h^
LS-LR-FeA-20H-20C	61.2 ± 7.7 ^a^	13.7 ± 0.5 ^de^
LS-CT-1H-30C	35.9 ± 0.9 ^c^	13.5 ± 0.1 ^e^
LS-TG-1H-30C	42.4 ± 3.7 ^bc^	17.9 ± 0.3 ^a^

Notes: The statistical analysis is performed by column. The different letters (a–h) indicate significant differences among samples (*p* < 0.05).

## Data Availability

The original contributions presented in the study are included in the article/Supplementary Materials, further inquiries can be directed to the corresponding author.

## Supplementary Materials

The following supporting information can be downloaded at: https://www.mdpi.com/article/10.3390/foods14111976/s1, Figure S1: SDS-PAGE profile of soy protein isolate (SPI), lupin flour (LF), and lupin and soy (LS) mixture under the most favourable cross-linking conditions of laccase (LR) and transglutaminase (TG). Lane 1: Protein marker; Lane 2: SPI; Lane 3: LF; Lane 4: LS-CT-1H-20C; Lane 5: LS-LR-1H-20C; Lane 6: LS-LR-FeA-1H-20C; Lane 7: LS-CT-20H-20C; Lane 8: LS-LR-20H-20C; Lane 9: LS-LR-FeA-20H-20C; Lane 10: LS-CT-1H-30C; Lane 11: LS-TG-1H-30C. CT—Control (non-enzyme-treated); FeA—Ferulic acid-added; H—Treatment time (h); C—Incubation temperature (°C). LR-treated samples were incubated at 20 °C for 1 h or 20 h with 142.5 U of LR/100 mg protein, while TG-treated samples were incubated at 30 °C for 1 h with 1.25 U of TG/100 mg protein. ***Summary:*** *Optimal LR and TG treatments confirmed successful cross-linking, with dark aggregates at the wells and disappearance of monomer bands.*
