# Supplementary material for: Optimising Enzymatic Cross-Linking: Impact on Physicochemical and Functional Properties of Lupin Flour and Soy Protein Isolate
Source: Foods. 2025 Jun 3;14(11):1976. doi: 10.3390/foods14111976 (PMC12155482; doi:10.3390/foods14111976)
Supplement: Supplementary file 1 [file foods-14-01976-s001.zip › foods-3583000-supplementary.pdf]

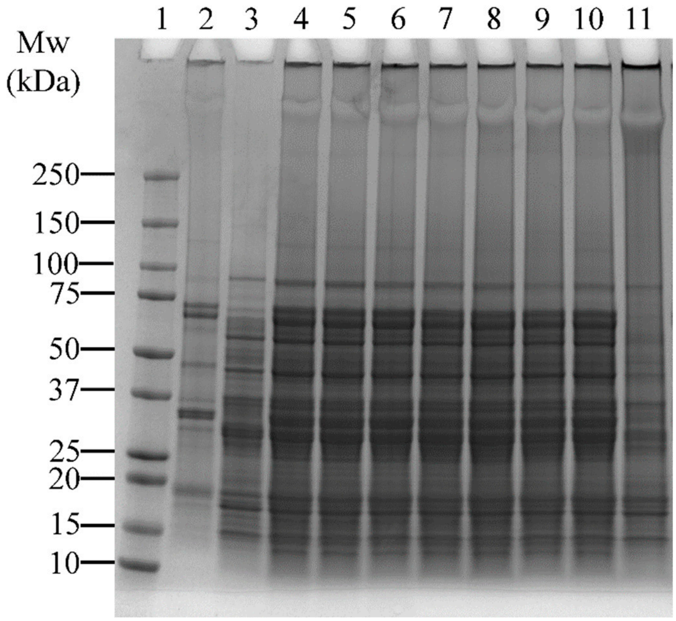

Figure S1. SDS-PAGE profile of soy protein isolate (SPI), lupin flour (LF), and lupin and soy (LS) mixture under the most favourable cross-linking conditions of laccase (LR) and transglutaminase (TG). Lane 1: Protein marker; Lane 2: SPI; Lane 3: LF; Lane 4: LS-CT-1H-20C; Lane 5: LS-LR-1H-20C; Lane 6: LS-LR-FeA-1H-20C; Lane 7: LS-CT-20H-20C; Lane 8: LS-LR-20H-20C; Lane 9: LS-LR-FeA-20H-20C; Lane 10: LS-CT-1H-30C; Lane 11: LS-TG-1H-30C. CT—Control (non-enzyme-treated); FeA—Ferulic acid-added; H—Treatment time (h); C—Incubation temperature (°C). LR-treated samples were incubated at 20 °C for 1 h or 20 h with 142.5 U of LR/100 mg protein, while TG-treated samples were incubated at 30 °C for 1 h with 1.25 U of TG/100 mg protein. ***Summary:*** *Optimal LR and TG treatments confirmed successful cross-linking, with dark aggregates at the wells and disappearance of monomer bands.*
